# Role of Transcranial Doppler Sonography in Diagnosis of Brain Death: A Single Center Study

**Published:** 2016

**Authors:** Seyed Mohammadreza Hashemian, Hosein Delavarkasmaei, Katayoun Najafizadeh, Meysam Mojtabae, Seyed Hossein Ardehali, Mohammad Reza Kamranmanesh, Niloofar Basharzad, Fariba Ghorbani

**Affiliations:** 1 Clinical Tuberculosis and Epidemiology Research Center, National Research Institute of Tuberculosis and Lung Diseases (NRITLD), Shahid Beheshti University of Medical Sciences, Tehran, Iran; 2 Department of Neurology, Shahid Beheshti University of Medical Sciences, Tehran, Iran; 3 Lung Transplantation Research Center, NRITLD, Shahid Beheshti University of Medical Sciences, Tehran, Iran; 4 Department of Critical Care, Shohadaye-Tajrish Hospital, Shahid Beheshti University of Medical Sciences; 5 Department of Anesthesiology & Critical Care and Pain Medicine, Shahid Beheshti University of Medical Sciences, Tehran, Iran; 6 Department of Pulmonology and Intensive Care Medicine, Shahid Labbafinejad Hospital, Shahid Beheshti University of Medical Sciences, Tehran, Iran; 7 Tracheal Diseases Research Center, NRITLD, Shahid Beheshti University of Medical Sciences, Tehran, Iran.

**Keywords:** Brain death, Transcranial Doppler Sonography, Electroencephalography

## Abstract

**Background:**

Diagnosis of brain death relies on clinical and electroencephalographic (EEG) criteria. Waiting for 24 hours is mandatory to make definitive diagnosis of the condition in the Iranian protocol. Although it has been previously shown that oscillatory or spiked systolic or reversed diastolic flow patterns in transcranial Doppler sonography (TCD) are associated with faster brain death confirmation, it has not yet been approved in our protocol. Thus, the aim of this study was to assess the applicability of this method to our organ donation system.

**Materials and Methods:**

This study was performed in Masih Daneshvari Organ Procurement Unit from July to December 2009. TCD from the middle cerebral and basilar arteries was attempted in 35 patients who fulfilled the clinical and EEG criteria for brain death. Extensive skull defects and hypotension (blood pressure < 80 mmHg) were the exclusion criteria. Examinations were made for about 30 minutes via temporal and occipital windows as soon as possible after diagnosis of brain death.

**Results:**

The mean age of cases was 31.9±14.78 years and 18 (51.4%) were males. The most prevalent cause of brain death was trauma (in 19 or 54.2% of cases). We were unable to detect any intracranial artery in 2 (5.7%) cases. There were no false negative or false positive results in the remaining ones. Detected ultrasonic patterns of cerebral vascular flow were systolic spike and oscillating signal in 29 (87.9%) and 4 (12.1%) donors, respectively.

**Conclusion:**

Our study showed that TCD results in brain dead cases were concordant with clinical and EEG criteria. Therefore, TCD, as a confirmatory test, can be applied for rapid diagnosis of brain death.

## INTRODUCTION

Diagnosis of brain death is a vital step in organ donation process both medically and legally. Accordingly, 88% of countries have decided to design a certain specific protocol for diagnosis of this condition (1). In this context, after initial diagnosis of brain death, definitive confirmation is of great importance. In some countries, clinical examination indicative of absence of brain stem reflexes is sufficient while in most others these findings have to be confirmed by accurate diagnostic tests (2). The choice of confirmatory test is specific to every country (3–6). Electroencephalography, TCD and somatosensory evoked potentials (SEP) are the most common examples (4, 6, 7). However, choosing the right test for definitive diagnosis has been a point of controversy and false positive and negative results have been rarely compared in different studies (3).

In Iran, two separate EEG waves below the 3 μV amplitude are necessary for diagnosis and flat EEG along with physical examination is the basis of diagnosis.

Since the techniques of recording Doppler signals of cerebral vascular flow were first described by Aaslid et al. in 1981 (8), this non-invasive method has obtained a great position in diagnosis of brain death. This method is not influenced by brain death resembling conditions such as barbiturate toxicity, hypothermia or some metabolic irregularities, making it superior to EEG in rapid brain death diagnosis (9).

Massive damage and necrosis of brain cells along with concomitant inflammation cause intolerable increase of intracranial pressure (ICP). When the ICP equals the diastolic arterial pressure, the brain is supplied only in systole and with additional increase of ICP over the systolic arterial pressure, cerebral perfusion will cease. Due to elasticity of the arterial wall and the compliance of the vasculature distal to the recording site, such cerebral circulatory arrest is associated with Doppler evidence of oscillatory movement of blood in the large arteries at the base of the brain. However, the net forward flow volume remains zero. As inflammation proceeds, the oscillations decrease in amplitude of spectral spikes until no pulsations are detectable (8, 10, 11). Cardiac arrest experience showed that 10–15 minutes of completely disabled cerebral blood flow equals irreversible death of brain tissue; therefore, the same period of characteristic blood flow seen in TCD is diagnostic for brain death.

To assess applicability of this method instead of EEG, this study evaluates different patterns of Doppler waves of cerebral arteries in brain dead cases.

## MATERIALS AND METHODS

This study was conducted in organ procurement unit of Masih Daneshvari Hospital. The TCD (SHINOVA EMS-9P system) using a 2 MHz PW probe was attempted in 35 cases that fulfilled the clinical and EEG criteria for brain death (12). All cases were comatose with Glaskow Coma Scale (GCS) of 3. There were no brain stem reflexes, including oculovestibular, oculocephalic, corneal, occulomotor, gag and cough reflexes. Atropine and apnea tests and 2 separately performed EEGs by a 6-hour interval were additional diagnostic tests preceding TCD. Cases with the following conditions were excluded:

1- Doppler sonography of cerebral arteries was impossible due to severe skull injury.
2- Severe edema of cerebral tissue which disrupted the window for evaluation of arteries.
3- Systolic blood pressure, less than 100 mmHg or bradycardia less than 60 beat/minute.

Donors’ data including age, sex and cause of brain death were recorded. The TCD waves were monitored for about 30 minutes for each patient via temporal window for middle cerebral artery (MCA) and occipital window for basilar artery as soon as possible after diagnosis of brain death. Oscillating flow or systolic spikes in addition to reversed diastolic flow were considered as indicatives of brain death.

## RESULTS

The mean age of 35 brain dead cases was 31.9±14.78 years (range 6–64 years) and 18 (51.4%) of them were males. Causes of brain death were as follows: Car accident trauma in 19, six donors had brain death due to anoxia following cardiopulmonary resuscitation (CPR), primary cerebral tumor in three, AV malformation in three and cardiovascular accident in two cases. Drug toxicity was also the underlying cause in two cases; 24 TCDs were performed by intensivists (68.5%) while others were done by neurologists (31.5%). Table 1 shows donors’ demographics and portions of TCD performing specialists.

**Table 1.** Donors’ demographics and portion of TCD performing specialists

		**Number**	**Percentage**
**Gender**	Male	18	51.4%
	Female	17	48.6%
**Brain death cause**	Trauma	19	54.2%
	Post CPR	6	17.1%
	Primary brain tumor	3	17.1%
	AV malformation	3	8.5%
	Cardiovascular accidents	2	5.7%
	Drug toxicity	2	5.7%
**TCD performer**	Intensivist	24	68.5%
	Neurologist	11	31.5%
**Total**		35	100%

No intracranial arteries were detected in two (5.7%) cases and actually 94.3% of TCDs were of diagnostic value. The typical ultrasonic patterns of cerebral flow cessation was systolic spike in 29 (87.8%) and oscillating signal in four (12.2%) of cases. In addition, reversed blood flow in diastolic phase was observed in four (12.2%) brain dead donors.

There were no false negative or false positive results in the remaining 33 ones. The TCD in two brain dead patients showed typical signs of brain death after two attempts while in the remaining one case, after four attempts we found ultrasonographic signs of brain death.

## DISCUSSION

This study showed that TCD is a reliable test for diagnosis of brain death. In 94.3% of donors, TCD was diagnostic; in 91.4% of which it was achieved on first attempt.

Reversed diastolic wave patterns were found to be prevalent in another study (7); however, we detected them in only 4 (12.1%) cases. This proportion can be due to the time interval, passed from brain circulation arrest in our cases that was not considered in the latter study. Generally, factors influencing wave patterns are as follows in the literature: the degree of bone damage in sides, asymmetric brain injury, elasticity of cerebral arteries, arterial pressure, administration of vasopressors and respiratory cycle (13,14).

Clinical criteria seem to be sufficient for diagnosis of brain death, but when it comes to organ donation, ethical and legal concerns necessitate performance of paraclinical tests, among which evaluation of cerebral blood flow is more important (7,15). The critical point is that test specificity for diagnosis of brain death is 100%(8).

Usually, the last signs of brain death consist of disappearance of ineffective spontaneous breathing and positive apnea test, as a result in some cases TCD is postponed until evidence of the 2 signs unveil (8, 12). Therefore, in 16.67% of our cases TCD was not performed at all.

According to many studies such as Ropper et al. (14) Davalos et al. (16) and Petty et al. (17), specificity of TCD has been 100%, however, it has also been reported between 91.3 and 100% based on the number of patients and the elapsed time from brain death event; therefore, the application of this test is associated with a great certainty for the diagnosis (10).

Dosemeci et al. (18) emphasized that by TCD repetition, sensitivity of diagnosis increased. TCD was repeated for diagnosis of three brain dead cases in our study as well. It has been previously shown that there is a short interval between cerebral blood flow cessation and appearance of brain death signs (19). In some cases, initial test result is false negative, but after a short time interval, complete picture of brain death appears. Because TCD performance is usually postponed until assumption of clinical criteria, false positive cases have not been observed.

In fact, the time between cerebral blood flow cessation and appearance of brain death pattern is less than 24 hours (11) based on which some researchers have recommended TCD to monitor patients with decreased level of consciousness. On the other hand, in false negative cases, repeated TCD leads to diagnosis (20). Oppositely, some false negative results have been reported in which the result did not change by repetition of TCD. This might be due to drainage of brain ventricles and subsequent discontinuation of ICP rising. In such cases, despite flat EEG and clinical signs of brain death, the results of TCD are not indicative of cerebral blood flow cessation.

As shown in previous studies, 10–20% of patients have poor window for TCD; in our study two (5.7%) cases had the same situation. Therefore, performer of this procedure has to be expert, since the result of this test is observer dependent (8, 21).

In most countries, it is a rule to consider a period of six to 72 hours from observing the first signs of brain death to make the definitive diagnosis (9). One of the reasons for small number of deceased organ donations in Iran might be inaccurate or delayed diagnosis of brain death in which EEG has flattened due to hypothermic effect, some metabolic disturbances or administration of CNS depressing drugs. As a result, possible brain death assessment in such patients has to be delayed until elimination of the mentioned conditions or clearance of the responsible drugs. Surprisingly, some adverse events or medical mismanagement in this period may cause severe damage to organs making them inappropriate for transplantation. This expresses the possible value of TCD in the whole organ donation and transplantation process in the future. Therefore, it is recommended to consider TCD performance for such cases in critical decision making situations.
